# European Research Council-funded grant: development of a novel cardiac tissue model

**DOI:** 10.1093/eurheartj/ehae740

**Published:** 2025-01-16

**Authors:** Gerardina Ruocco, Daniele Testore, Valeria Chiono

**Affiliations:** 1 Department of Mechanical and Aerospace Engineering, Politecnico di Torino, Corso Duca Degli Abruzzi 24, 10129 Turin, Italy; 2 Interuniversity Center for the Promotion of the 3Rs Principles in Teaching and Research, Centro 3R, Italy

Heart diseases (HDs) represent a major health challenge, being the leading cause of mortality and morbidity worldwide.^1^ HDs impose a huge economic burden to the healthcare system and the whole society, which is expected to increase due to the growth and aging of the population.^2^ Hence, safe and effective therapies for HD treatment are highly demanded. However, their development has been mostly limited by pre-clinical testing in poorly predictive *in vitro* 2D cell cultures and *in vivo* animal models affected by interspecies differences.^3^ Limitations in pre-clinical testing also impose safety risks for patients involved in the next clinical trials, which may include impaired cardiac function at different levels up to fatal arrhythmias, ischaemic events, myocardial infarction, and injuries to cardiac valves, the conduction system and the pericardium. In other cases, patient-specific cardiotoxicity risks have been detected only after drug approval causing their withdrawal from the market.^4^ Notably, drugs developed for other purposes than HD treatment (e.g. chemotherapeutics) also need early cardiotoxicity risk assessment as they may have adverse effects on the heart.

In this context, the design and validation of patient-specific methods for the *in vitro* testing of cardiac safety could accelerate new drug development, reduce the use of experimental animals and mitigate potential harms to humans.

## *In vitro* assessment of drug cardiotoxicity: achievements and challenges

The primary goal of safety evaluation is the timely identification of the arrhythmogenic potential of drug candidates. Different *in vitro* cell assays providing complementary information have been approved by the regulatory agencies, making use of 2D cell cultures, such as^5^:

- The Human Ether-a-go-go-related Gene (hERG) channel assay to study the inhibition of potassium ion channel that can lead to prolongation of QT interval (i.e., the total time from ventricular depolarization to complete repolarization) and potentially fatal arrhythmias.
- Electrophysiological assays to study the action potentials and ion channel currents by patch-clamp techniques.
- Mitochondrial function assays to study mitochondrial membrane potential and the production of reactive oxygen species and adenosine triphosphate.
- Contractility assays to assess cardiomyocyte functionality through video-based motion analysis or impedance-based assays.

However, the choice of cell sources and the culture conditions are fundamental to get predictive outcomes from *in vitro* tests. In the last decade, human-induced pluripotent stem cells-derived cardiomyocytes (hiPSC-CMs) have emerged as promising cell candidates for the *in vitro* assessment of drug cardiotoxicity, due to their availability, close molecular and functional properties to primary human cardiomyocytes and patient-specificity.^6^ The comprehensive *in vitro* ProArrhythmia initiative validated the use of hiPSC-CM monolayers for the assessment of the proarrhythmic potential of new drugs, through non-invasive label-free monitoring of their electrophysiological properties by multielectrode array technology.^7^ However, the immature foetal- or neonatal-like phenotype of iPSC-CMs is a key limitation for reliable prediction of *in vivo*-like pharmacological responses from *in vitro* drug testing.

Organ-on-chips and tissue engineering solutions, integrating multiple *in vivo*-like biophysical and biochemical cues favour hiPSC-CM maturation.^8,9^ Examples include the *in vitro* culture of hiPSC-CMs on scaffolds with cardiac tissue-like composition, stiffness, functionality (e.g., electroconductivity), architecture (i.e. mimicking cardiac tissue anisotropy), and the application of external mechanical and/or electrical stimulations.^8,9^

However, previously developed cardiac tissue models have been limited by: (i) a complex design, requiring specific technological skills for their implementation and affecting interlaboratory reproducibility; (ii) low throughput; and (iii) lack of an integrated biosensing system for the detection of cell electrophysiological properties.

Hence, one key issue for effective *in vitro* pre-clinical testing of drugs and advanced therapies is the lack of easy-to-use, robust, high-throughput technologies for the engineering, and real-time electro-physiological assessment of mature human cardiac tissue models.

## EMPATIC platform: an engineered solution for human cardiac tissue modelling and testing

**Figure 1:**
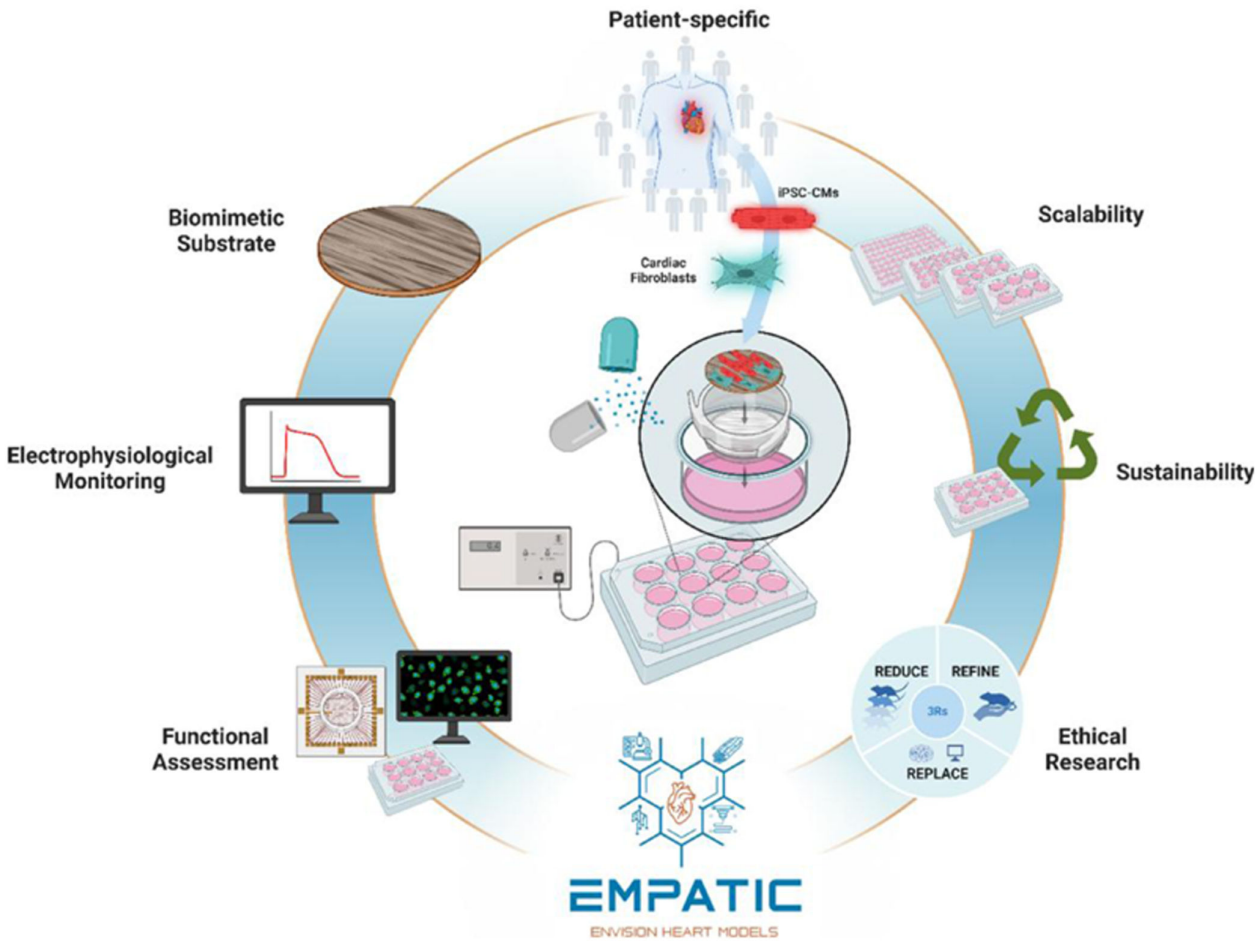
EMPATIC platform: an engineered solution for human cardiac tissue modelling and testing. Funded by the ERC Proof of Concept Grant (ERC-2023-POC), EMPATIC aims at developing an easy-to-use and versatile system for the *in vitro* modelling of mature human cardiac tissue from human induced pluripotent stem cells-derived cardiomyocytes and cardiac fibroblasts cultured on biomimetic scaffolds providing multiple biochemical and biophysical stimuli to cells. It enables live monitoring of cell electrophysiological properties; furthermore, platform design is compatible for micros-copy and/or advanced functional analysis. Other key advantages include: user-friendliness, high-throughput, sustainability (80% reusable components), and versatility for the testing of drugs, nanomedicines, and advanced therapies (created with BioRender.com)

To overcome such major limitations, the European Research Council (ERC) Proof of Concept Grant (ERC-2023-POC) EMPATIC (‘Engineered multiwell-plate platforms integrating biochemical and biophysical cues for the functional maturation and electrophysiological monitoring of cardiac tissue models’; grant agreement 101158332) develops a novel user-friendly and versatile multiwell-plate platform, integrating advanced bioengineering tools, for the *in vitro* modelling of mature functional human cardiac tissue from hiPSC-CMs, co-cultured with human cardiac fibroblasts, and the non-invasive label-free monitoring of electrophysiological properties (Figure 1). Specifically, the platform is equipped with biomimetic scaffolds, imparting biochemical and biophysical cues to cells, and specific bioelectronic components for both electrical stimulation during cell culture and biosensing for the detection of cell functionality (Figure 1).

EMPATIC has several advantages:

- Simplicity of use: it is a ready-to-use advanced platform for the *in vitro* engineering of human cardiac tissue.
- High-throughput: it is compatible for use in commercial multiwell plates with different formats.
- Sustainability: the platform is biodegradable and produced avoiding the use of toxic solvents; 80% of plastic components can be reused upon cleaning and sterilisation.
- Versatility of use: it provides cardiac tissue models allowing the testing of compounds, nanomedicines and advanced therapies (e.g. cell therapies).
- Modularity: the engineered cardiac tissues, once ready, can be easily handled and are compatible with microscopy analysis and advanced functional assessment.

EMPATIC project provides a robust, predictive, high-throughput, reproducible, and versatile platform for the *in vitro* modelling of human cardiac tissue and cardiotoxicity assessment of drugs, nanomedicines, and advanced therapies. EMPATIC platform may also find application in basic research, such as the study of the functional maturation of cardiomyocytes from other sources than hiPSC-CMs (e.g., directly reprogrammed fibroblasts^10^) or other cells (e.g., neurons and skeletal muscle cells).

EMPATIC project is co-ordinated by Prof. Valeria Chiono from Department of Mechanical and Aerospace Engineering at Politecnico di Torino, with consolidated expertise in tissue engineering, and under the scientific collaboration with Prof. Giacinto Luigi Cerone and Prof. Alberto Botter from Department of Electronics and Telecommunication and LISiN Laboratory at Politecnico di Torino, with recognized bioelectronics know-how. Starting in July 2024 and running over 18 months, EMPATIC project provides a translational approach, envisioned to impact researchers from universities, research institutes, and R&D divisions of pharma/biotech companies, developing drugs and advanced therapies for applications in cardiac regenerative medicine and other fields (e.g. cancer treatment).

## Conclusion and future developments

In conclusion, EMPATIC bio-convergence approach, deriving from an interdisciplinary cooperation among scientists with complementary expertise in biology, chemistry, physics, bioelectronics, tissue engineering, and other fields, will provide advanced tools for the engineering of *in vitro* human functional cardiac tissue models through an easy-to-use modular platform.

EMPATIC project contributes to the design of predictive methods for early pre-clinical drug assessment: their integration in drug validation process by the European Medicines Agency (EMA) and Food and Drugs Administration (FDA) will progressively reduce the use of experimental animals (Directive 2010/63/EU), improve pre-clinical testing relevance and human safety, and provide predictive *in vitro* platforms for personalized drug screening.

In the future, artificial intelligence applied to EMPATIC platform will allow to correlate drug inputs (drug dose, number, and frequency of administrations, etc.) with tissue structure and function in order to set patient-specific drug treatments.
